# Anomalous left circumflex artery: Implications for valve-sparing root replacement

**DOI:** 10.1016/j.xjtc.2021.01.031

**Published:** 2021-01-30

**Authors:** Naoto Fukunaga, Mark D. Peterson

**Affiliations:** Division of Cardiovascular Surgery, Unity Health Network and Department of Surgery, St Michael's Hospital, University of Toronto, Toronto, Ontario, Canada

**Figure:**
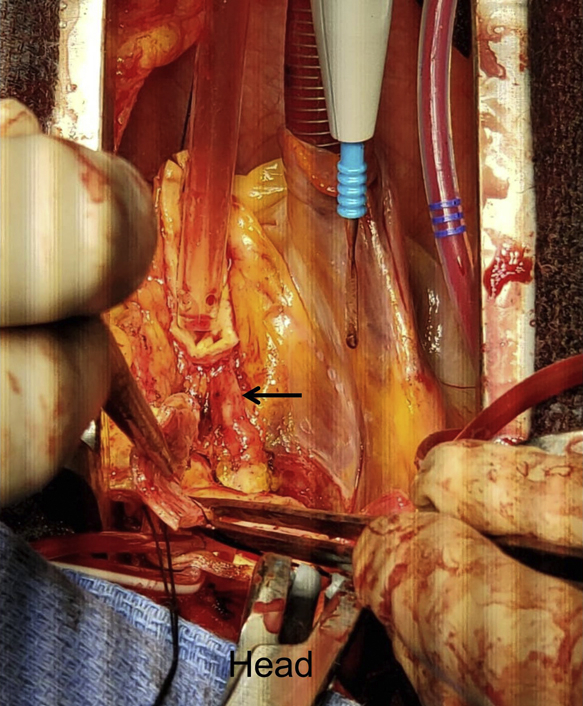
Retroaortic anomalous left circumflex artery originating from the right coronary artery.

**Central Message**

Careful consideration of coronary anomalies during aortic root surgery is important in selecting the surgical approach and avoiding coronary artery injury.

See Commentaries on pages 149 and 151.

Anomalous origin of the left circumflex artery (LCX) has been recognized in 0.67% of patients who have undergone selective coronary angiography.^1^ We present our case to emphasize the implications of an anomalous LCX when performing valve-sparing root replacement.

## Case Description

**Figure 1:**
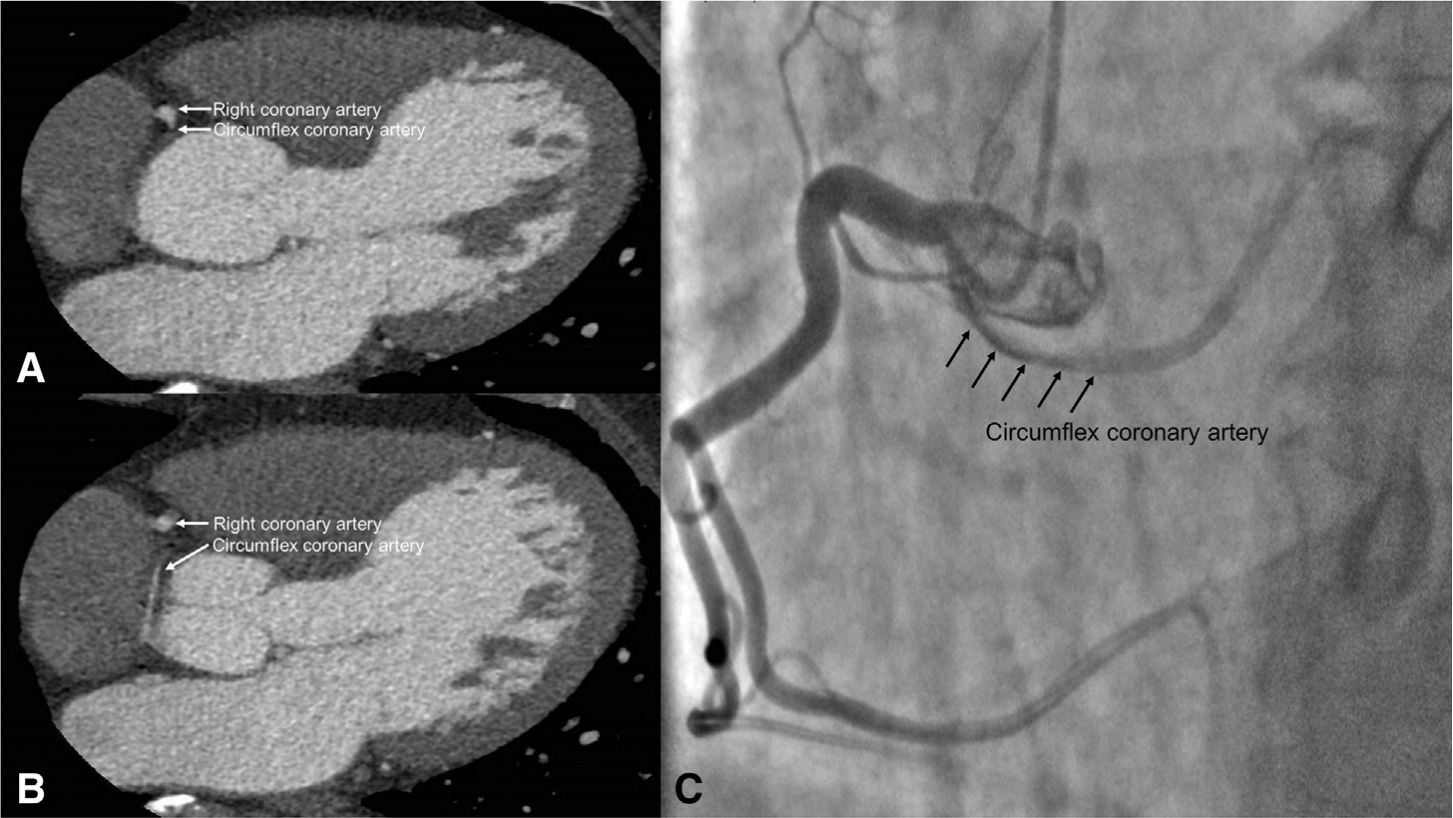
A, Contrast computed tomography showing the right coronary artery and a retroaortic anomalous left circumflex artery originating from the right coronary artery. B, The circumflex coronary artery courses in close proximity and inferior to the annulus of the noncoronary sinus. The diameter of aortic root measures 53 mm. No calcification is observed in the aortic valve. C, Coronary angiography demonstrating an anomalous left circumflex artery originating from the right coronary artery (*arrows*). A nonobstructive stenosis in the left circumflex artery is noted. The right coronary is dominant and free of obstructive coronary artery disease.

A 64-year-old man with a history of hypertension presented with a 2-year history of shortness of breath. He denied exertional chest discomfort. He did not have features suggestive of a syndromic aortopathy and had no family history of aortic disease. Transthoracic echocardiography showed moderate to severe aortic regurgitation with a severely dilated left ventricle and preserved left ventricular function and an annular diameter of 2.5 cm. The aortic valve was tricuspid. Contrast computed tomography scan demonstrated an anomalous LCX originating from the right coronary artery with a retroaortic course (Figure 1, *A* and *B*), an ascending aortic aneurysm of 6.0 cm, and a dilated aortic root of 5.3 cm. Coronary angiography confirmed an anomalous LCX originating from the dominant right coronary artery, coursing into the obtuse marginal distribution (Figure 1, *C*).

**Figure 2:**
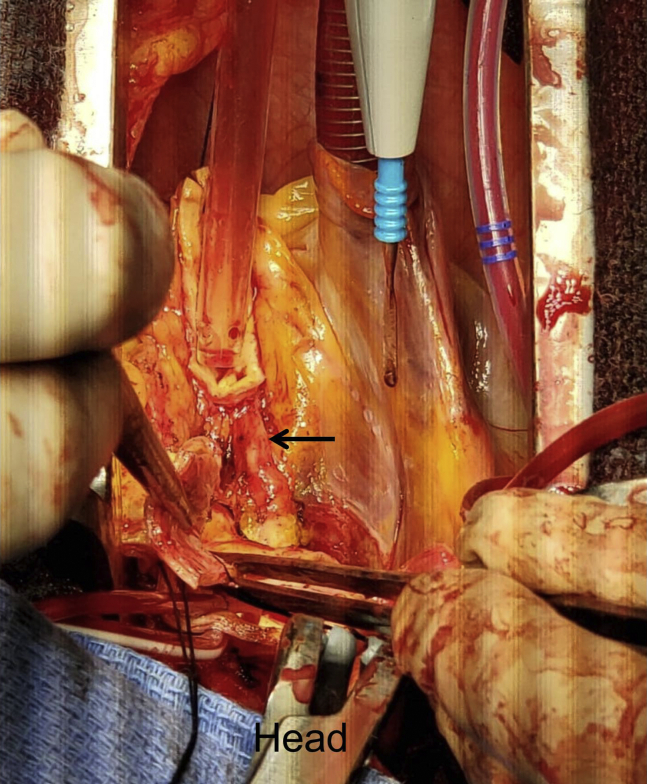
At surgery following creation of a coronary button, the arrow indicates an anomalous left circumflex artery coming off the right coronary artery, coursing inferior to the noncoronary sinus. The anomalous circumflex is shown after it is dissected away from the inferior margin of the annulus of the noncoronary sinus (*arrow*). A selective cardioplegia cannula was inserted into the right coronary ostium to protect the right and left circumflex territories.

The patient was brought to the operating theater. Cardiopulmonary bypass was established via proximal aortic arch and right atrial cannulation, and systemic cooling was initiated targeting a nasopharyngeal temperature of 24°C. Following aortic cross-clamping and cardiac arrest achieved by selective cardioplegic perfusion to the left main and right coronary ostium, the aortic root was prepared by resecting the aortic root tissue with a 5-mm cuff and mobilizing the coronary artery buttons. The anomalous LCX was identified at its origin of the right coronary button coursing down toward the noncoronary sinus of Valsalva (Figure 2 and Video 1). The close association between the aortic annulus and the anomalous LCX would have resulted in compression or suture impingement.

Because the aortic annulus was not dilated, we decided to perform a remodeling root replacement to keep the sutures above the annular plane and avoid injury to the anomalous LCX. A 28-mm woven polyester graft was fashioned into 3 tongues and was sutured to the aortic wall remnant with 4-0 polypropylene to create 3 neosinuses. The aortic cusp height was measured using a Schafer caliper, and all 3 cusps had an effective height of >9 mm. Both coronary buttons were reimplanted in a standard fashion. Before completing the aortic root repair, we performed a hemiarch reconstruction under moderate hypothermic circulatory arrest and antegrade cerebral perfusion via the innominate artery.^2^

Postoperative echocardiography confirmed excellent aortic valve function with no aortic insufficiency and prolapse. The perioperative electrocardiogram was unchanged.

## Discussion

Current management strategies for anomalous aortic origin of coronary artery are based on the probability of sudden cardiac death or myocardial infarction and the presence of symptoms.^3^ Identification of the subtype and the natural history is relevant during aortic root surgery to prevent coronary artery injury.

Several case series have been published describing aortic valve replacement or aortic root surgery in patients with an anomalous LCX.^4^, ^5^, ^6^ The presence of an anomalous LCX during aortic valve replacement or root surgery could lead to injury by suture ligation, compression by a prosthetic valve, injury during resection of the noncoronary sinus of Valsalva or suturing for sinus repair, and distortion of reimplanted coronary artery. Mobilization of an anomalous LCX away from aortic annulus is mandatory to avoid serious complications. Coronary artery bypass grafting was an option to mitigate the effect from potential injury or compression.^5^ Our patient denied angina and did not have significant obstructive coronary disease. Therefore, we felt preserving that the natural anatomy was preferable to adding a bypass graft.^3^

The main advantage of the remodeling procedure is that it allowed suturing of the graft to the aortic cuff above the aortic annulus, avoiding injury to the anomalous LCX. The aortic annulus measured 25 mm, the upper limits of normal for a male with a body surface area of 2.0 m^2^. We believed that the risk of LCX injury would have been elevated by adding an annular stabilization procedure. Had annular stabilization been indicated, we would have performed a suture annuloplasty using an expanded polytetrafluorethylene suture, tied around a Hegar dilator.^7^^,^^8^ We believe this technique would have allowed an annuloplasty with minimal risk of injury to the anomalous LCX. We would add this type of annuloplasty for annular dimensions exceeding 26-27 mm.^6^

Successful aortic root surgery in the presence of an anomalous coronary artery must consider the anatomic relationship between the course of the aberrant artery and the aortic annulus on preoperative imaging modalities, to plan the operative strategy and prevent arterial injury.^3^^,^^9^

## Supplementary Data

Video 1 Intraoperative video showing the relationship between an anomalous left circumflex artery and a noncoronary annulus. Video available at: https://www.jtcvs.org/article/S2666-2507(21)00114-0/fulltext.
