# Supplementary figures and images for: Anomalous left circumflex artery: Implications for valve-sparing root replacement
Source: JTCVS Tech. 2021 Jan 30;7:146–8. doi: 10.1016/j.xjtc.2021.01.031 (PMC8311588; doi:10.1016/j.xjtc.2021.01.031)

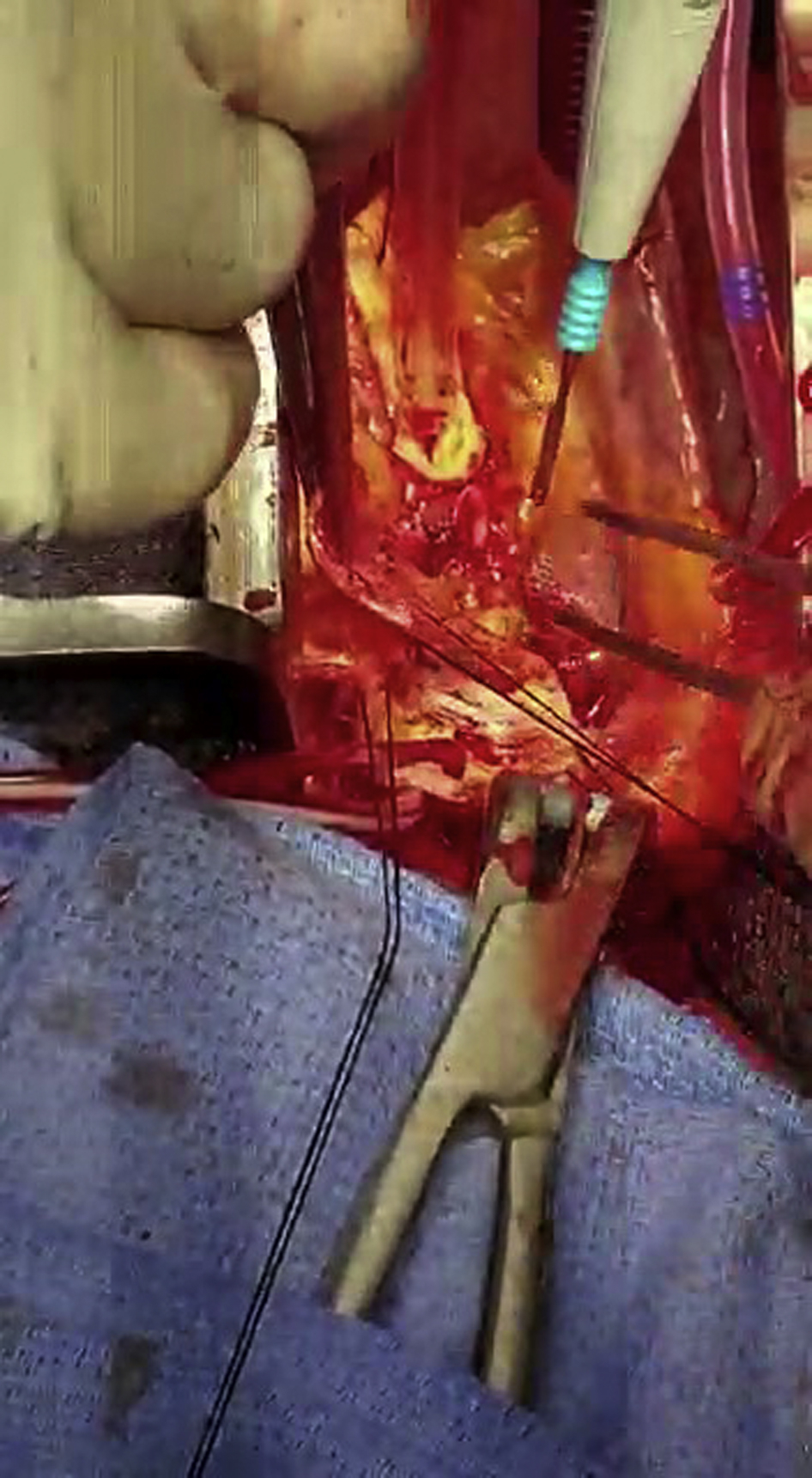

Supplement: Video 1 — Intraoperative video showing the relationship between an anomalous left circumflex artery and a noncoronary annulus. Video available at: https://www.jtcvs.org/article/S2666-2507(21)00114-0/fulltext. [file fx2.jpg]
